# Metampicillin is a cyclic aminal produced by reaction of ampicillin with formaldehyde

**DOI:** 10.1038/s41598-020-74990-1

**Published:** 2020-10-21

**Authors:** Raphael Reinbold, Tobias John, Paolo Spingardi, Akane Kawamura, Christopher J. Schofield, Richard J. Hopkinson

**Affiliations:** 1 Chemistry Research Laboratory, 12 Mansfield Road, Oxford, OX1 3TA UK; 2 School of Natural and Environmental Sciences, Chemistry Bedson Building, Kings Road, Newcastle Upon Tyne, NE1 7RU UK; 3 Leicester Institute of Structural and Chemical Biology and School of Chemistry, University of Leicester, Henry Wellcome Building, Lancaster Road, Leicester, LE1 7RH UK

**Keywords:** Chemical genetics, Chemical modification

## Abstract

Metampicillin is a β-lactam antibiotic that is prepared by the reaction of ampicillin with formaldehyde. Although metampicillin has been studied for treatment of infections in animals and humans, its structure has been unclear. We report NMR studies revealing that metampicillin contains a formaldehyde-derived cyclic aminal. NMR time-course experiments with excess formaldehyde in solution show formation of another product with an additional exocyclic hemiaminal group formed by reaction with the cyclic aminal nitrogen. The exocyclic hemiaminal group is readily removed by reaction with the formaldehyde scavenger 1,3-cyclohexanedione, whereas the cyclic aminal methylene exhibits greater stability. The overall results assign the structure of metampicillin as containing a cyclic aminal and further reveal the potential for complexity in the reaction of formaldehyde with biomedicinally relevant molecules.

## Introduction

Metampicillin is a β-lactam antibiotic approved for use in human medicine as recognised by the World Health Organization^1^, and which is also used in veterinary medicine according to the European Medicines Agency^2^. It is prepared by the reaction of ampicillin (**1**) and formaldehyde (HCHO)^3^. Metampicillin is considered to be a prodrug that releases **1** after acid-catalysed fragmentation in the stomach. Metampicillin accumulates in bile^4^ and is reported to be more stable in serum than in aqueous acidic conditions^5^. Whilst in most infections the clinical efficacy of metampicillin is comparable to that of **1**, its accumulation in bile leads to greater efficacy in treatment of biliary infections^6^. Biliary concentrations of parenterally injected metampicillin are 300 times higher than concentrations of **1** administered under the same conditions, suggesting that the liver selectively secretes metampicillin over **1**^4^.

**Figure 1:**
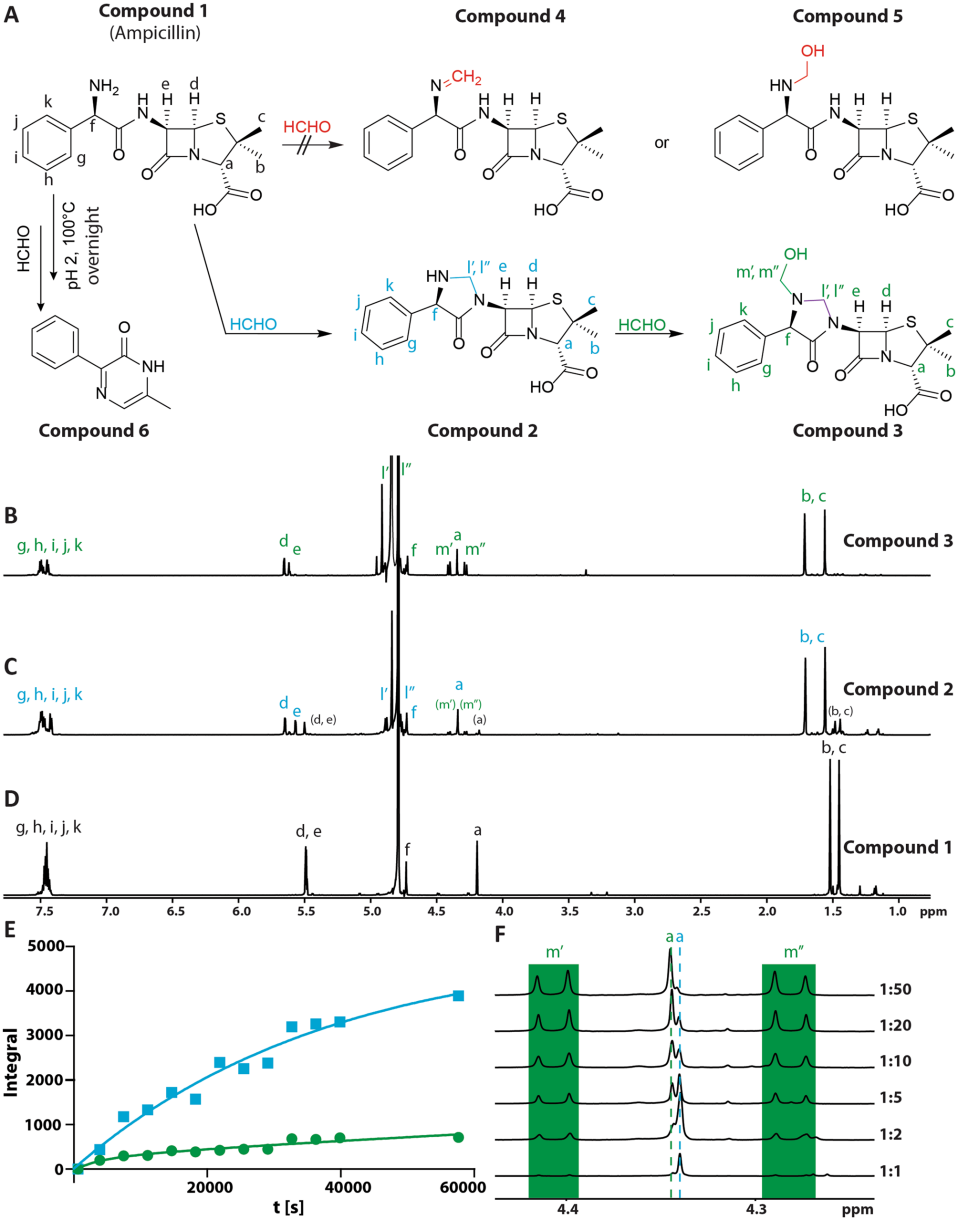
| Ampicillin (**1**) reacts with formaldehyde (HCHO) to form cyclic aminals (**2** and **3**). (**A**) Previously proposed structures for metampicillin (**4** and **5**) and the metampicillin structure (**2**) identified in this work by NMR after HPLC purification/lyophilisation. Compound **3** is observed in solution but could not be isolated. **6** is obtained by reaction of **1** with HCHO in acidic conditions (100 °C) as reported^9^. **B/C/D.** ^1^H (700 MHz) spectra of **1** (D), **2** (C) and **3** (B) in D_2_O. (**C**) Adding a twofold excess of HCHO to **1** results in **2**; trace amounts of **1** and **3** are observed (in brackets). (**D**) In the presence of excess HCHO, further reaction to give **3** occurs. Structures were assigned using 2D NMR (Figs. S3, S5 and S6). (**E**) Plot of integrals of the cyclic aminal resonance of **2** (blue) and the hemiaminal resonance of **3** (green) observed during reaction of **1** with a twofold excess of HCHO. (**F**) Magnified section of the ^1^H NMR spectra showing both hemiaminal protons (green, “m”) of **3** after reaction of **1** with different amounts of HCHO (4 h). The two overlapping singlets at δ_H_ 4.34 ppm are assigned to the CHCO_2_H hydrogens of **2** (higher field singlet, blue “a”) and **3** (lower field singlet, green “a”). Addition of further HCHO correlates with increased **3** and decreased **2**.

Given that metampicillin has been studied for use in human biliary infections, it is surprising that its structure has not been unequivocally assigned. The presence of the core ampicillin-derived scaffold of **1** is accepted; however, the structure of its HCHO-derived component(s) is contentious (**2–5**; Fig. 1A). Both imine^7^ (**4**) and hemiaminal^8^ (**5**) structures for metampicillin have been proposed, and there are other possibilities (**2**, **3**; Fig. 1A). Studies on the stability of the HCHO-derived component(s) of metampicillin are also lacking, though ampicillin (**1**) is reported to form 3-phenyl-6-ethyl-pyrazin-2-one with HCHO under acidic conditions (**6**, Fig. 1A)^9^.

We report NMR studies on the structure and stability of metampicillin in water. The results confirm the presence of the core scaffold of **1** in metampicillin and identify co-existing cyclic aminal and hemiaminal products in solution. The cyclic aminal is more stable and likely represents the major component of solid metampicillin (**2**).

## Results

Initial studies focused on determining the structure of purified metampicillin after synthesis from **1** and HCHO. The preparation of metampicillin was adapted from reported procedures, in which **1** was reacted with an excess of HCHO in water^8^. Thus, the sodium salt of **1** (37 mg) was dissolved in water and reacted with an excess of HCHO (10-fold) for 2 h at room temperature. The resultant mixture was then purified by reversed-phase HPLC (Fig. S1). The major product was subjected to lyophilisation to give a white solid.

NMR analysis of the solid in D_6_-DMSO (Fig. S2) supported the presence of the β-lactam core ring structure (CHS and COCHNCO, δ_H_ 5.62–5.53 ppm; see SI for numbering and full assignment). The resonances at δ_H_ 4.36, δ_H_ 1.65 and δ_H_ 1.49 were assigned to the remaining penicillin ring-derived hydrogens on the basis of 2D ^1^H-^13^C-HSQC and ^1^H-^13^C-HMBC correlations (Fig. S3). These resonances and the aromatic ^1^H resonances at δ_H_ 7.44–7.39 ppm, δ_H_ 7.38–7.33 ppm, and δ_H_ 7.31–7.26 are very similar to those in the ^1^H NMR spectrum of **1** (Fig. 1D). ^1^H resonances at δ_H_ 4.82 ppm and δ_H_ 4.52 ppm, however, did not correlate with similar resonances in the spectrum of **1** and were therefore proposed to correspond to HCHO-derived protons; 2D ^1^H-^13^C HSQC analysis (Fig. S3) suggested these protons are attached to the carbon at δ_C_ 63.0 ppm (assigned to C-10), implying formation of a methylene group. ^1^H-^13^C HMBC correlations of δ_H_ 4.82 ppm with δ_C_ 60.9 ppm, and of δ_H_ 5.57 ppm with δ_C_ 63.0 ppm (Fig. S3) suggest formation of a cyclic aminal (**2**, Fig. 1A + C). Such a structure has not been proposed for metampicillin but was the only HCHO-derived product detectable after HPLC purification and lyophilisation under our conditions (Figs. 1A, S2 and S3).

We then conducted NMR time-course analyses on the reaction of **1** and HCHO in water. Initially, the reaction of **1** with a 5-fold excess of HCHO was monitored in D_2_O over 11 h at room temperature (Fig. S4). ^1^H resonances corresponding to the cyclic aminal of **2** (methylene at δ_H_ 4.88 ppm and δ_H_ 4.77 ppm) were observed at the first time-point (30 min) and reached a maximum intensity after 3 h. New lower-level ^1^H resonances, tentatively assigned to a hemiaminal, were also observed (δ_H_ 4.41 ppm and δ_H_ 4.28 ppm) which increased in intensity over time. To promote formation of the new species (**3**) and to enable NMR characterisation, a sample was prepared with a 10-fold excess of HCHO and reacted overnight. Under these conditions, the new species (**3**) was the major product. ^1^H-^1^H-COSY and ^1^H-^13^C-HSQC analyses on the mixture enabled assignment of the ^1^H resonances to the previously assigned cyclic aminal (δ_H_ 4.90 and 4.72 ppm) and, importantly, a novel HCHO-derived hemiaminal (**3**) (δ_H_ 4.40 and 4.27 ppm) (Figs. S5 and S6). ^1^H-^13^C-HMBC correlations between the hemiaminal (δ_H_ 4.40 ppm and δ_H_ 4.27 ppm) and both the cyclic aminal (δ_C_ 64.3 ppm) and the carbon attached to the side chain α-amino group (δ_C_ 63.7 ppm) suggest that the hemiaminal and aminal co-exist and are connected to the side-chain amine (**3**, Figs. 1A + B and S6; see SI for numbering and full assignment).

Time-course studies were then conducted at different HCHO concentrations (Fig. S7). When equimolar amounts of HCHO were added to **1**, ^1^H resonances corresponding to the cyclic aminal (**2**) were observed at early timepoints (Fig. S8), whilst those for hemiaminal (**3**) were only observed at low intensity suggesting that cyclic aminal (**2**) forms prior to **3** under limiting HCHO. When a 2-fold excess of HCHO was added (Fig. 1E), cyclic aminal resonances corresponding to **2**, as well as cyclic aminal and hemiaminal resonances corresponding to **3** were observed. The cyclic aminal resonances of **2** appeared rapidly and were present at a higher intensity throughout the time-course. Therefore, formation of the cyclic aminal-containing **2** appears most efficient under these conditions; further reaction of **2** with HCHO to form the cyclic aminal- and hemiaminal-containing **3** then occurs. The amount of **3** observed was increased at higher HCHO concentrations (Figs. 1F, S7 and S9). Repeating the time-course analyses under alkaline conditions (pD 9) also revealed formation of **3**; under these conditions, reaction to give **3** was faster, presumably due to the increased nucleophilicity of the side-chain amine (Fig. S10).

The stability of the cyclic aminal and hemiaminal groups in **2** and **3** were then investigated using the HCHO scavenger 1,3-cyclohexanedione^10^. 1,3-cyclohexanedione reacts with HCHO to form quasi-stable hemiaminal and dimeric adducts^10^. Reaction mixtures containing **1** and a 2-fold excess of HCHO were prepared in D_2_O and reacted for 24 h at room temperature. Varying amounts of 1,3-cyclohexanedione were then added and the mixtures were transferred to an NMR tube and monitored by ^1^H NMR and ^1^H-^13^C-HSQC. When 1 or 2 equivalents of 1,3-cyclohexanedione were added, the hemiaminal group was readily removed from **3** to give **2** (Fig. S11A); however, the cyclic aminal group of **2** was unaffected. With 4 equivalents of 1,3-cyclohexanedione, the aminal ^1^H resonances of **2** decreased to 40% intensity after 14 h (Fig. S11B), while complete loss of the aminal **2** was observed after 10 min when exposed to 20 equivalents of 1,3-cyclohexanedione (Figs. S11C and S12). Overall, these findings imply the hemiaminal group in **3** is labile but that the cyclic aminal is significantly more stable.

## Discussion

NMR studies on the reaction of **1** and HCHO in aqueous solution reveal the formation of two products, i.e. **2** and **3**. We accrued no evidence for the previously proposed structures for metampicillin **4** and **5**, though these may be intermediates *en route* to **2** and **3** (Fig. 1A). We also did not observe formation of 3-phenyl-6-ethyl-pyrazin-2-one (**6**), which is formed by reaction of **1** and HCHO under prolonged acidic conditions (Fig. 1A)^9^. The results reveal that the cyclic aminal **2** is stable to lyophilisation whereas **3**, which is observed in solution with an excess of HCHO, is likely not. These findings illustrate the intricacies in the reactions of HCHO in reactions with drugs bearing nucleophilic groups such as ampicillin.

The structural insights provided here may be relevant to understanding why metampicillin appears to be selectively delivered to the bile compared to its parent ampicillin drug **1**^4^ and, more generally, in identifying pro-drug-type derivatives of antibacterials and other drugs that are targeted at specific organs or tissues^11^. Given the relative lack of toxicity of HCHO at low doses and its use in many cosmetics and pro-drugs (acyloxymethyl groups are often attached to therapeutics to improve their bioavailability and stability^12^), we suggest further work on using HCHO and related reactive carbonyl compounds in targeting drugs to specific tissues is of interest. Finally, the work provides further evidence for the potentially unique reactivity of the seemingly simple carbonyl compound HCHO with both small and large biologically relevant molecules. This can often occur in a manner that enables the reversible formation of cyclic structures. As exemplified in our work with ampicillin (**1**)/metampicillin (**2**), the HCHO-derived cyclic product can have different physiochemical properties to the parent drug in aqueous solution^13–15^.

## Methods

### Synthesis of metampicillin^8^

Ampicillin (**1**) sodium salt (37 mg, 0.1 mmol) was reacted with aqueous formaldehyde (1 ml of a 1 M solution, 1 mmol, 10 equivalents). To obtain an aqueous formaldehyde solution, paraformaldehyde was suspended in H_2_O and heated with a heatgun until a clear colourless solution was obtained^14^; the mixture was then stirred at room temperature for 2 h. The reaction mixture was subjected to HPLC purification (2% (v/v) MeCN (in H_2_O, 0.1% (v/v) aqueous formic acid) to 60% (v/v) over 12 min (reversed-phase column ACE5 C18, 100 × 21.2 mm, Hichrom)). The product was lyophilised to give a colourless solid, which was insoluble in D_2_O.

### In situ NMR studies

The ampicillin (**1**) sodium salt (18.5 mg, 0.05 mmol) was mixed with formaldehyde (500 μl of 1 M stock in D_2_O, 0.5 mmol, 10 equivalents) and reacted overnight at room temperature without adjusting the pD; analysis was by 2D NMR.

### Time-course studies

The ampicillin (**1**) sodium salt (8.7 mg, 23.3 µmol) was dissolved in D_2_O and the pD was adjusted to 7.4 and 9 (corresponding to pH 7 and 8.6) using NaOD and DCl (total volume: 1 ml). Paraformaldehyde was suspended in D_2_O and heated with a heatgun until a clear colourless solution was obtained; the pD was adjusted to 7.4 or 9 using NaOD and DCl and D_2_O added to a final HCHO concentration of 0.33 M. For time-course analyses, the ampicillin stock solution (100 μl, 2.33 μmol) at pD 7.4 or 9 and D_2_O (400 µl) was mixed and a ^1^H NMR spectrum (700 MHz) recorded. Subsequently, HCHO (10 equivalents, 23.3 μmol; pD 7.4 or 9) was added and the reaction was monitored for the indicated time. Further time-course analyses were conducted for the formation of metampicillin without adjusting the pD using ampicillin sodium salt (2.33 µmol) and different amounts of HCHO (from a 0.67 M stock in D_2_O) as described above to a final volume of 675 μl. A plot ^1^H NMR of integrals was made using GraphPad Prism Version 5.04, and a curve fit obtained using non-linear regression.

### Stability studies

The ampicillin (**1**) sodium salt (100 μl of a 23.3 mM stock of ampicillin in D_2_O, 2.33 µmol) was mixed with HCHO (7 μl of 0.67 M stock in D_2_O, 4.66 µmol, 2 equivalents) without adjusting the pD; D_2_O was added to a final volume of 675 μl. The reaction was allowed to proceed overnight at room temperature. 600 µl (4.12 μmol formaldehyde, 2.06 μmol ampicillin) of this mixture was then transferred to an NMR tube and different amounts of a 1 M 1,3-cyclohexanedione solution were added (1:1, 1:2, 1:4, 1:8, 1:20, with respect to the total amount of HCHO). The intensity of proton signals corresponding to the HCHO-derived products **2** and **3** was monitored by ^1^H NMR and 2D NMR (700 MHz, 400 MHz) over time.

## Supplementary information

Supplementary Information
