# Supplementary material for: Metampicillin is a cyclic aminal produced by reaction of ampicillin with formaldehyde
Source: Sci Rep. 2020 Oct 21;10:17955. doi: 10.1038/s41598-020-74990-1 (PMC7577985; doi:10.1038/s41598-020-74990-1)
Supplement: Supplementary file 1 — Supplementary Information [file 41598_2020_74990_MOESM1_ESM.pdf]

Supplementary Information

Metampicillin is a Cyclic Aminal Produced by Reaction of Ampicillin With Formaldehyde

Raphael Reinbold,[†a] Tobias John,[†a] Paolo Spingardi,[a] Akane Kawamura,[a,b] Christopher J. Schofield,*[a] and Richard J. Hopkinson*[a,c]

[a]Chemistry Research Laboratory, 12 Mansfield Road, Oxford, OX1 3TA, United Kingdom. [b]School of Natural and Environmental Sciences, Chemistry Bedson Building, Kings Road, Newcastle Upon Tyne, NE1 7RU. [c]Leicester Institute of Structural and Chemical Biology and School of Chemistry, University of Leicester, Henry Wellcome Building, Lancaster Road, Leicester, LE1 7RH, United Kingdom.

† These authors contributed equally.

Reagents

Sodium ampicillin was from Apollo Scientific; paraformaldehyde, NaOD, DCl and 1,3-cyclohexanedione were from Sigma-Aldrich.

Characterisation Methods

NMR spectra (HSQC, HMBC, COSY, ^{1}H, ^{13}C) were obtained using either Bruker Avance AV700, AV600, AV500, or AV400 spectrometers. The Bruker internal referencing procedure (edlock) was used to reference spectra to the solvent peak (δ_H = D_2O 4.79 ppm; δ_H = D_6-DMSO 2.5 ppm, δ_C = D_6-DMSO 39.52 ppm).[1] For reporting multiplicities, the following abbreviations are used: m (multiplet), q (quartet), d (doublet), s (singlet). For ^{1}H-^{13}C-HSQC and ^{1}H-^{13}C-HMBC spectra, the color codes are as follows: red HSQC signals denote CH or CH_3, blue HSQC signals denote CH_2 coupling, and green signals denote HMBC coupling. High-resolution mass spectra measurements (HRMS) were obtained using a Waters LCT Premier ESI mass spectrometer. Mass spectra were measured using an Agilent single quadrupole machine. Melting point measurements were conducted on a Stuart automatic melting point SMP40 with a heating rate of 20 °C per minute. Infrared (IR) spectra were measured using a Bruker Tensor 27 FT-IR spectrometer using attenuated total reflection (ATR) at room temperature.

Characterisation of HPLC purified metampicillin (2)

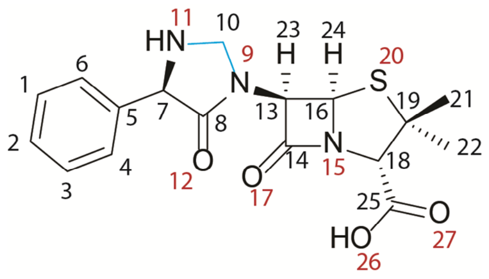

Using the purification procedure described in the Materials and Methods section, metampicillin (**2**) was obtained as a colorless solid (12 mg, 33 µmol, 33%).

^{1}H NMR (700 MHz, D_6-DMSO) δ 7.44 – 7.39 (m, 2H, 4, 6), 7.38 – 7.33 (m, 2H, 1, 3), 7.31 – 7.26 (m, 1H, 2), 5.62 – 5.53 (m, 2H, 23, 24), 4.82, 4.52 (ABq, J = 6.7 Hz, 2H, 10', 10''), 4.54 (s, 1H, 7), 4.36 (s, 1H, 18), 1.65 (s, 3H, 21 or 22), 1.49 (s, 3H, 21 or 22); ^{13}C NMR (101 MHz, D_6-DMSO) δ = 173.7 (C8), 171.2 (C14), 168.8 (C25), 138.4 (C5), 128.2 (C1, C3), 127.4 (C2), 127.2 (C4, C6), 70.2 (C18), 66.3 (C16), 64.7 (C19), 63.0 (C10), 60.9 (C7), 60.2 (C13), 30.5 (C21 or C22), 26.7 (C21 or C22); ESI-MS, $[M+H]^+$: 362.12 (calculated), 362.100 (observed); HRMS (ESI+) $[M+H]^+$: 362.1169 (calculated for $C_{17}H_{20}N_3O_4S$), 362.1169 (observed); IR ṽ 1768.84 cm^{-1}, 1737.55 cm cm^{-1} (CON) (see Figure S13); melting point: 195 °C (degradation).

Characterisation of *in situ* reaction product (3)

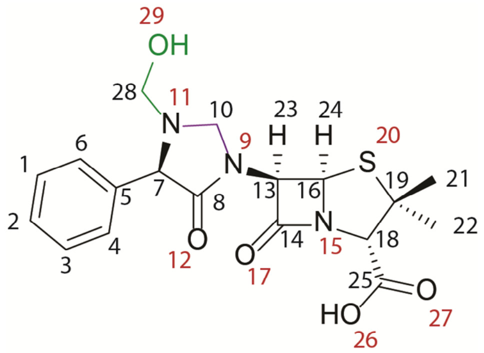

Compound **3** was prepared by reaction of **1** (ampicillin) with 10-fold excess HCHO in D_2O. It was characterised *in situ* using ^{1}H NMR and ^{1}H-^{13}C-HMBC/^{1}H-^{13}C-HSQC NMR.

^{1}H NMR (700 MHz, D_2O) δ 7.51 – 7.43 (m, 5H, 1, 2, 3, 4, 6), 5.66 (d, J = 3.9 Hz, 1H, 24), 5.62 (d, J = 3.9 Hz, 1H, 23), 4.90, 4.72 (ABq, J = 5.3, 2H, 10', 10''), 4.76 (s, 1H, 7), 4.40, 4.27 (ABq, J = 11.4, 2H, 28', 28''), 4.35 (s, 1H, 18), 1.72 (s, 3H, 21 or 22), 1.57 (s, 3H, 21 or 22); ^{13}C NMR (101 MHz, D_2O) δ = 174.0 (C8), 171.9 (C14), 171.9 (C25), 135.5 (C5), 129.0 (C1-4, C6), 71.4 (C28), 73.2 (C18), 65.6 (C16), 65.4 (C19), 64.3 (C10), 63.7 (C7), 60.0 (C13), 30.1 (C21 or C22), 26.7 (C21 or C22).

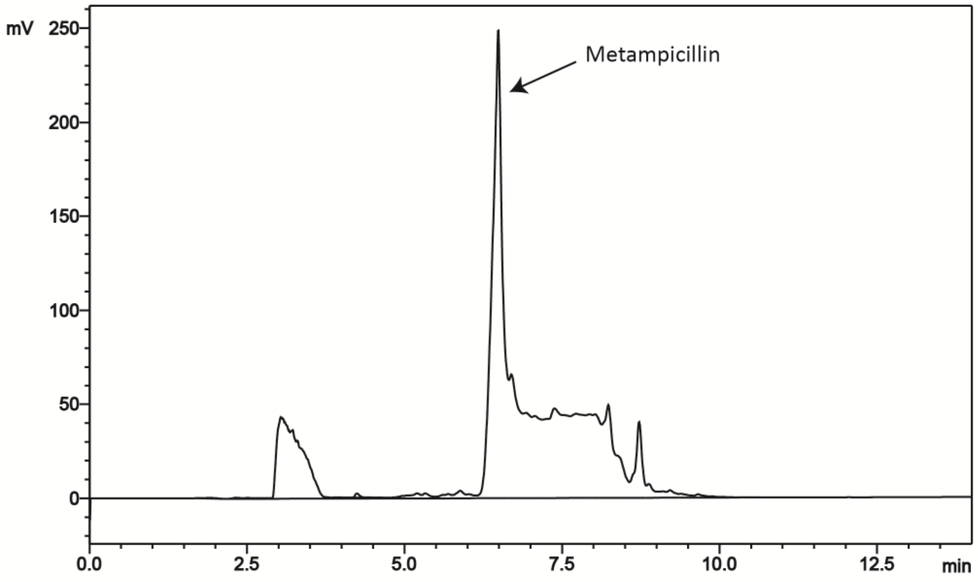

Figure S1 | HPLC analysis of the mixture obtained by reaction of ampicillin (**1**) and formaldehyde (HCHO). Fractions containing metampicillin (**2**) (as determined by LCMS) were combined and freeze-dried. See the Materials and Methods section for details.

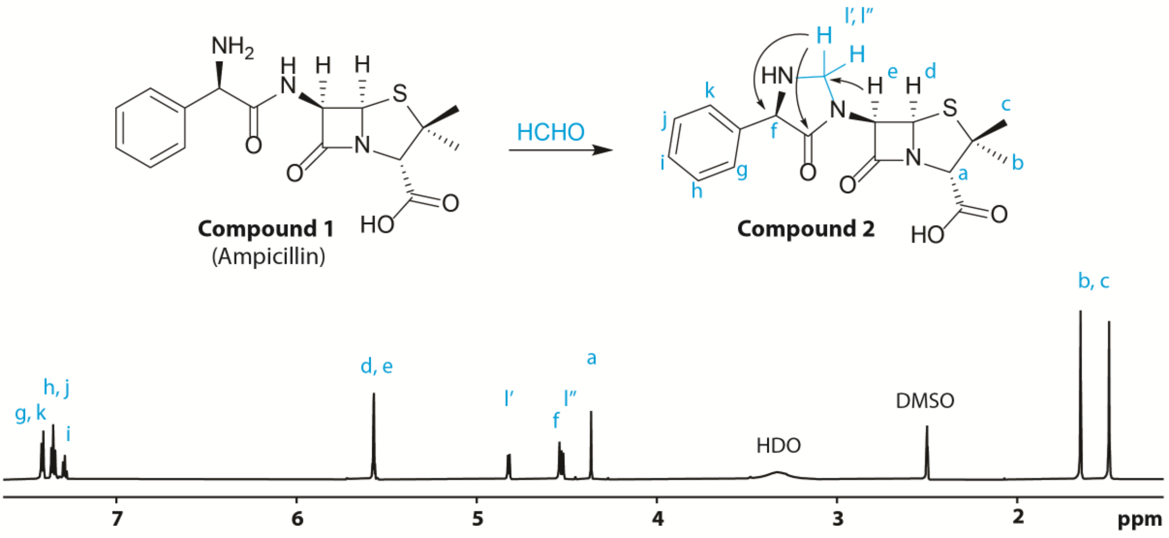

Figure S2 | ^{1}H NMR (700 MHz, D_6-DMSO) spectrum of HPLC purified metampicillin (**2**) prepared from **1**. ^{1}H-^{13}C HMBC correlations (indicated by arrows) of HPLC-purified metampicillin reveal a methylene bridge between the α-amino group and the amide nitrogen (see Figure S3).

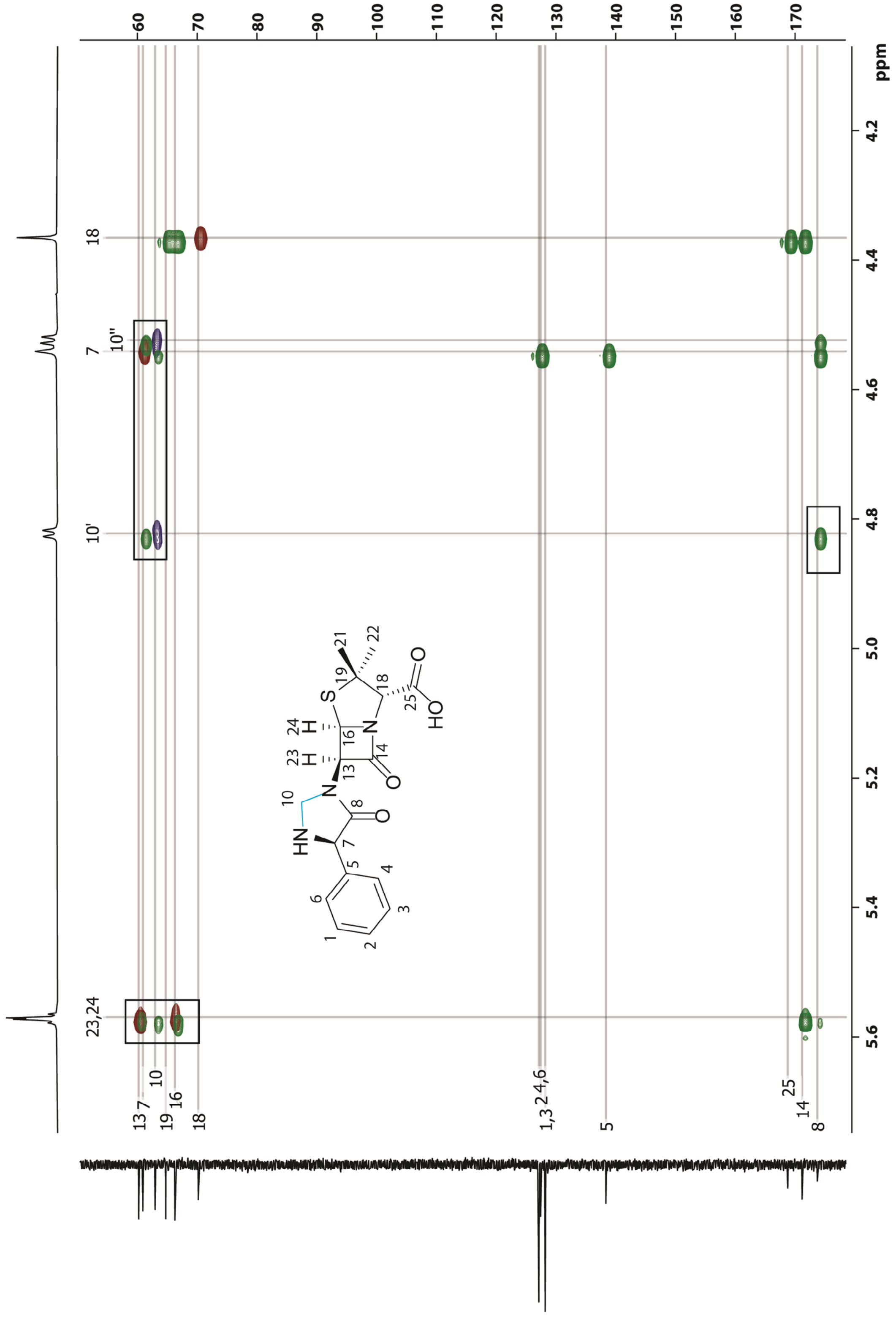

Figure S3 | ^{1}H-^{13}C-HMBC and ^{1}H-^{13}C-HSQC overlay of HPLC-purified metampicillin (**2**) in D_6-DMSO. Interactions important in the assignment are in boxes. The x-axis shows the ^{1}H (700 MHz) spectrum and the y-axis shows the ^{13}C spectrum.

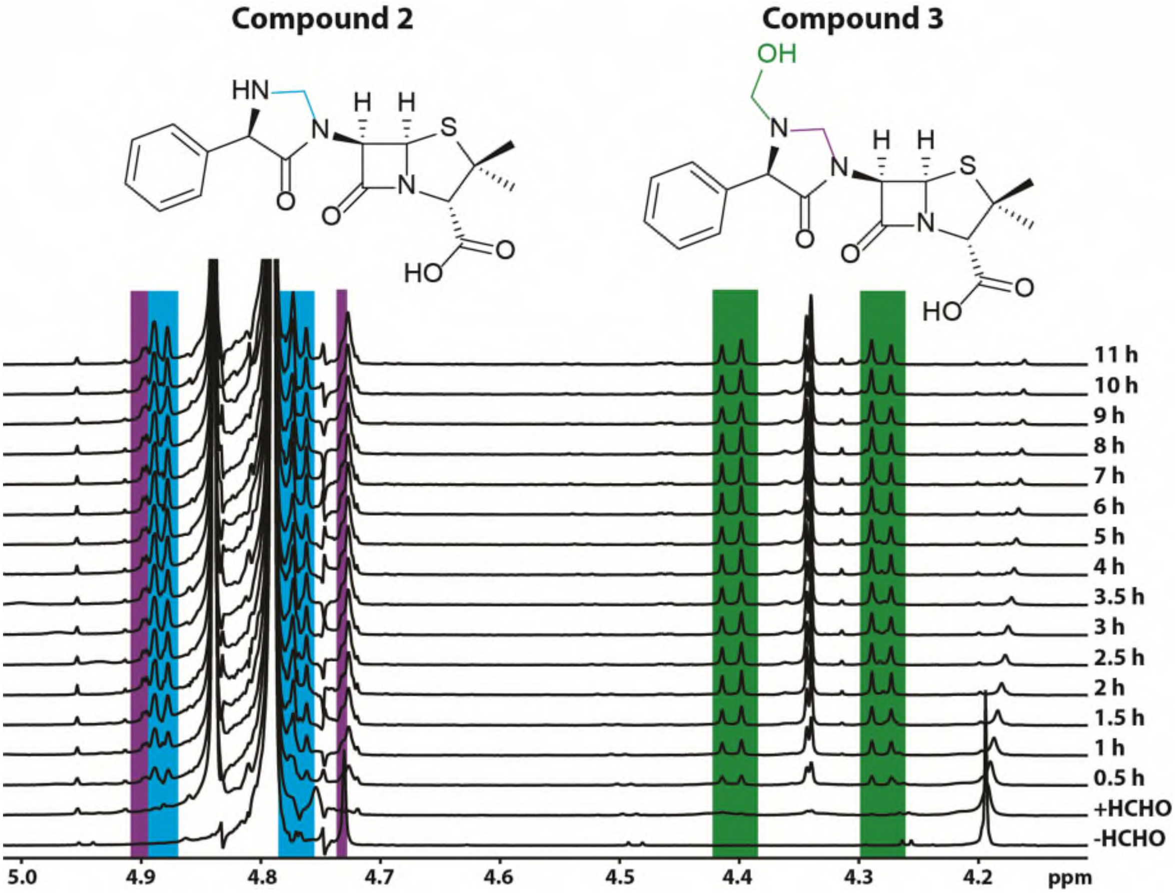

Figure S4 | Reaction of **1** with HCHO with monitoring by ^{1}H NMR (700 MHz, D_2O). Time-course analysis of reaction of **1** with a 5-fold HCHO excess shows the formation of a cyclic aminal (**2**, blue) and a product with an additional hemiaminal group (**3**, green). Note the shoulder (δ_H 4.89, purple) downfield to the cyclic aminal signal of **2** at δ_H 4.88 (blue) and the low-level signal at δ_H 4.73 (purple) correspond to the cyclic aminal methylene in **3**. Note the broad nature of the signals at δ_H 4.2 ppm, which implies a dynamic equilibrium on the NMR timescale under these conditions.

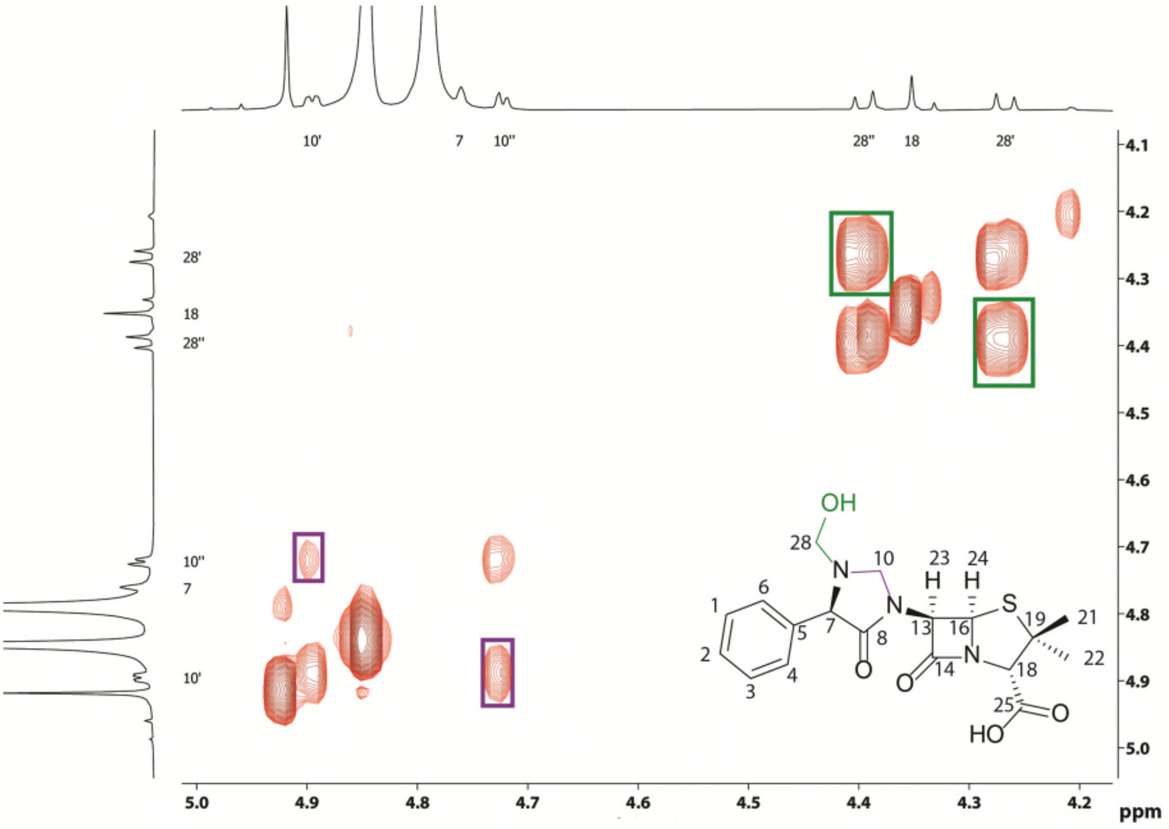

Figure S5 | NMR analysis of the *in situ* reaction product (**3**) resulting from mixing ampicillin with a 10-fold excess of HCHO in D_2O. ^1H-^1H-COSY analysis reveals a product with two additional methylene groups relative to the ampicillin (**1**) starting material. The hemiaminal signals are highlighted in green boxes and the cyclic aminal methylene signals are highlighted in purple boxes.

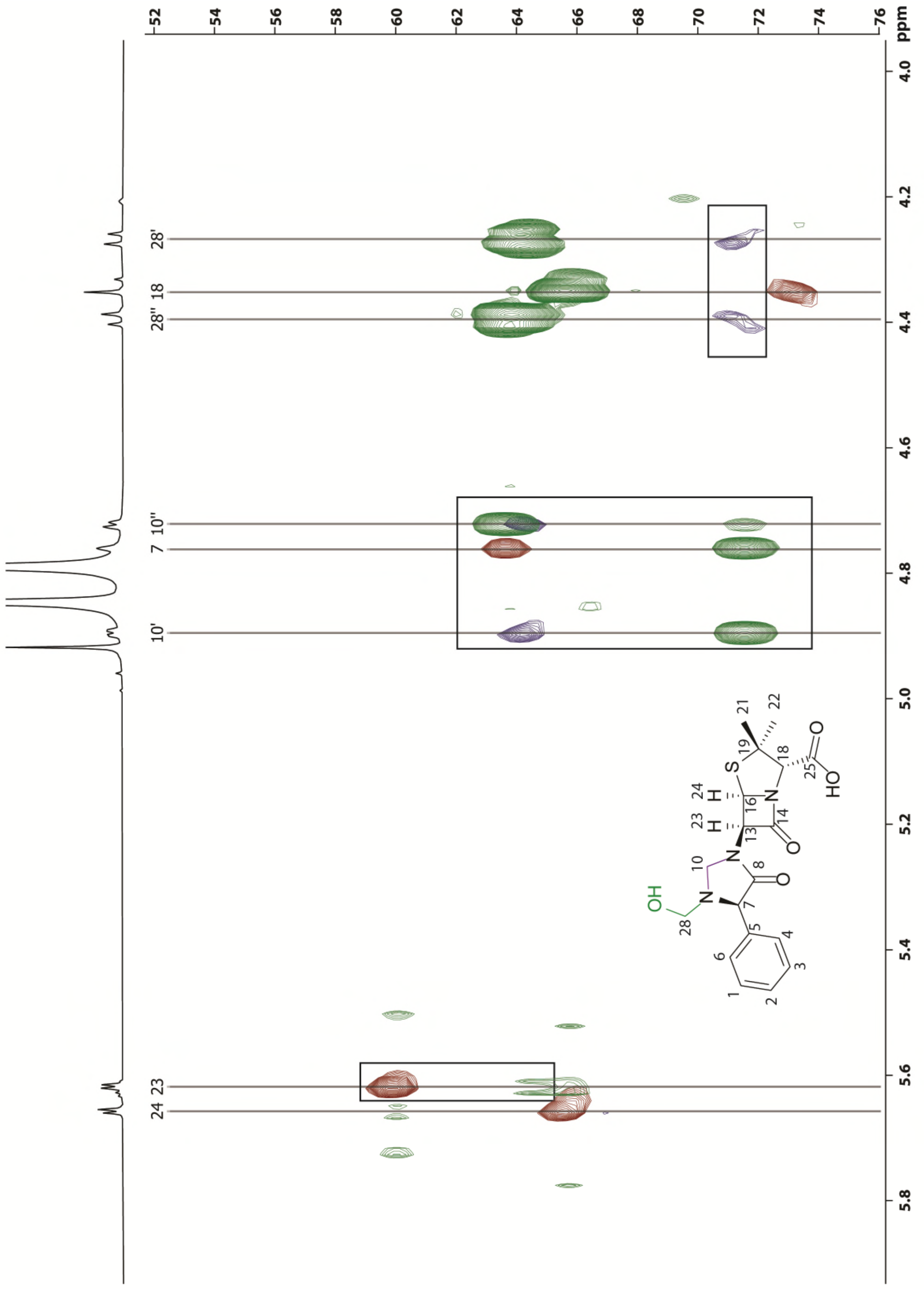

Figure S6 | ^{1}H-^{13}C-HMBC and ^{1}H-^{13}C-HSQC (methylene region) NMR analysis of the *in situ* reaction product (**3**) resulting from mixing ampicillin (**1**) with a 10-fold excess of HCHO in D_2O. Interactions important in the assignment are in boxes. The x-axis shows the ^{1}H (700 MHz) spectrum. Note that hydrogen 23 shows an HMBC correlation with C-10 (cyclic aminal methylene), but not with C-28 (hemiaminal methylene).

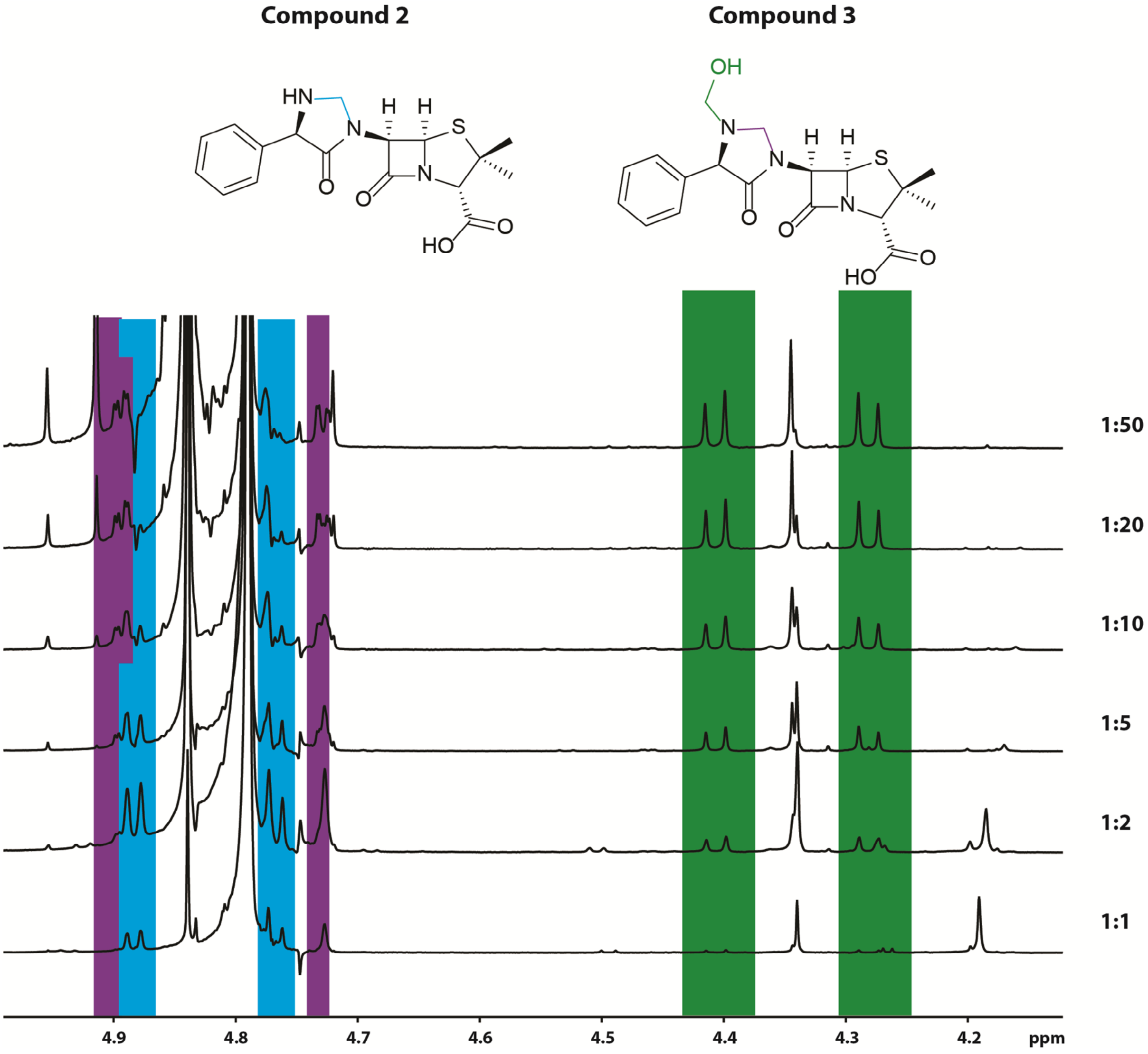

Figure S7 | ^{1}H NMR (700 MHz, D_2O) spectra of the cyclic aminal and hemiaminal regions after mixing of **1** with different amounts of HCHO (4 h). The two doublets in blue correspond to the cyclic aminal of **2**. The low-level signal shaded in purple at low concentrations of HCHO corresponds to the cyclic aminal of **3**. With increasing concentrations of HCHO, this signal increases in intensity while the cyclic aminal signal of **2** (blue) decreases in intensity. The hemiaminal signal of **3** is shaded in green. Close analysis of the spectra indicates the potential for further complexity. Not all the low intensity resonances were assigned, e.g. the 'β-lactam' proton region (Figure S6, δ_H 5.63 ppm). Further, 'doubling' of some resonances was observed. This notably occurs at the C-10 resonance of **3** (purple), but neither at methylenes of C-28 (green) nor at C-10 of **2** (blue). We speculate that these resonances may result from addition of a further HCHO to **3,** or, e.g., are possibly due to a HCHO-derived imine.

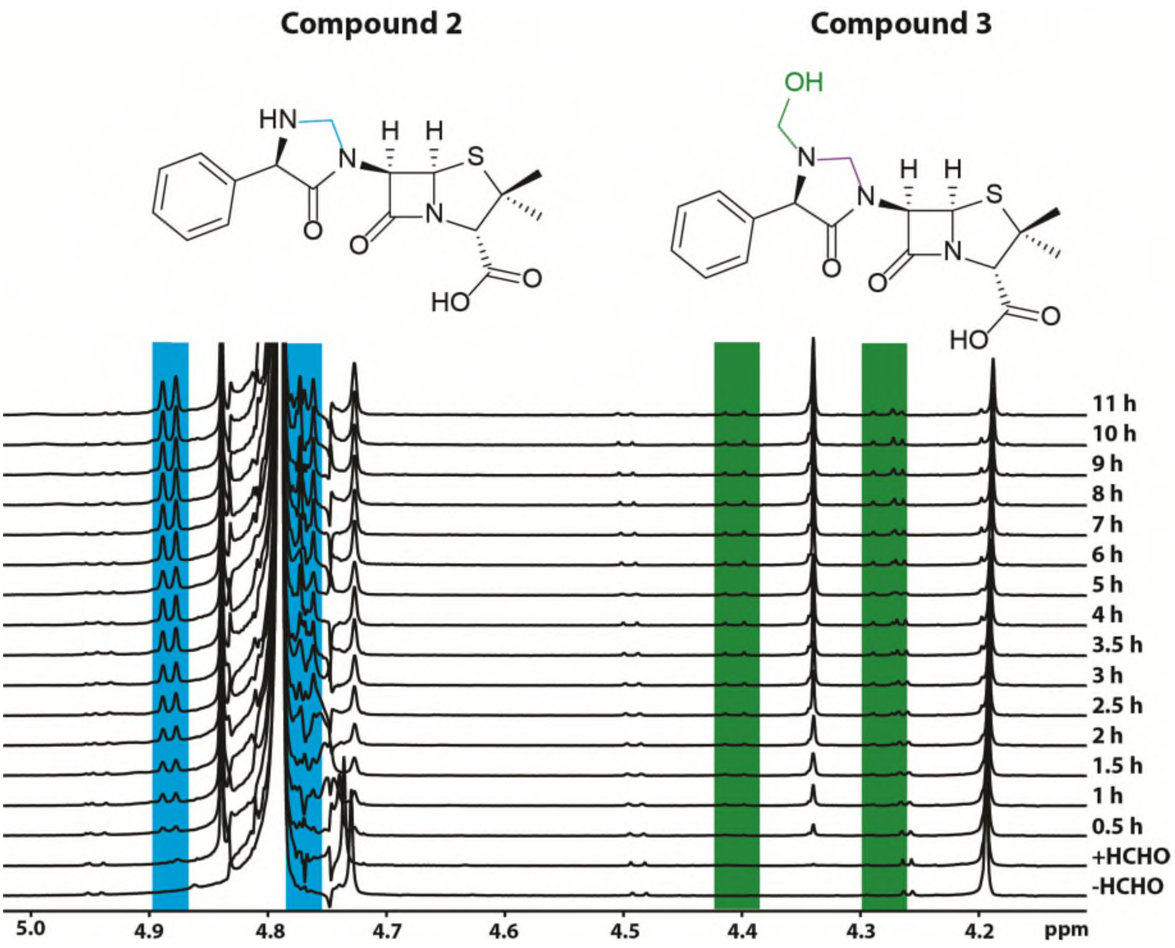

Figure S8 | Reaction of **1** with HCHO with monitoring by ^{1}H NMR (700 MHz, D_2O). Time-course of reaction of ampicillin with equimolar HCHO manifests formation of **2** as the major product (blue). Only trace amounts of **3** are observed (green). Note the cyclic aminal methylene protons of **3** were not observed, likely due to their low intensities.

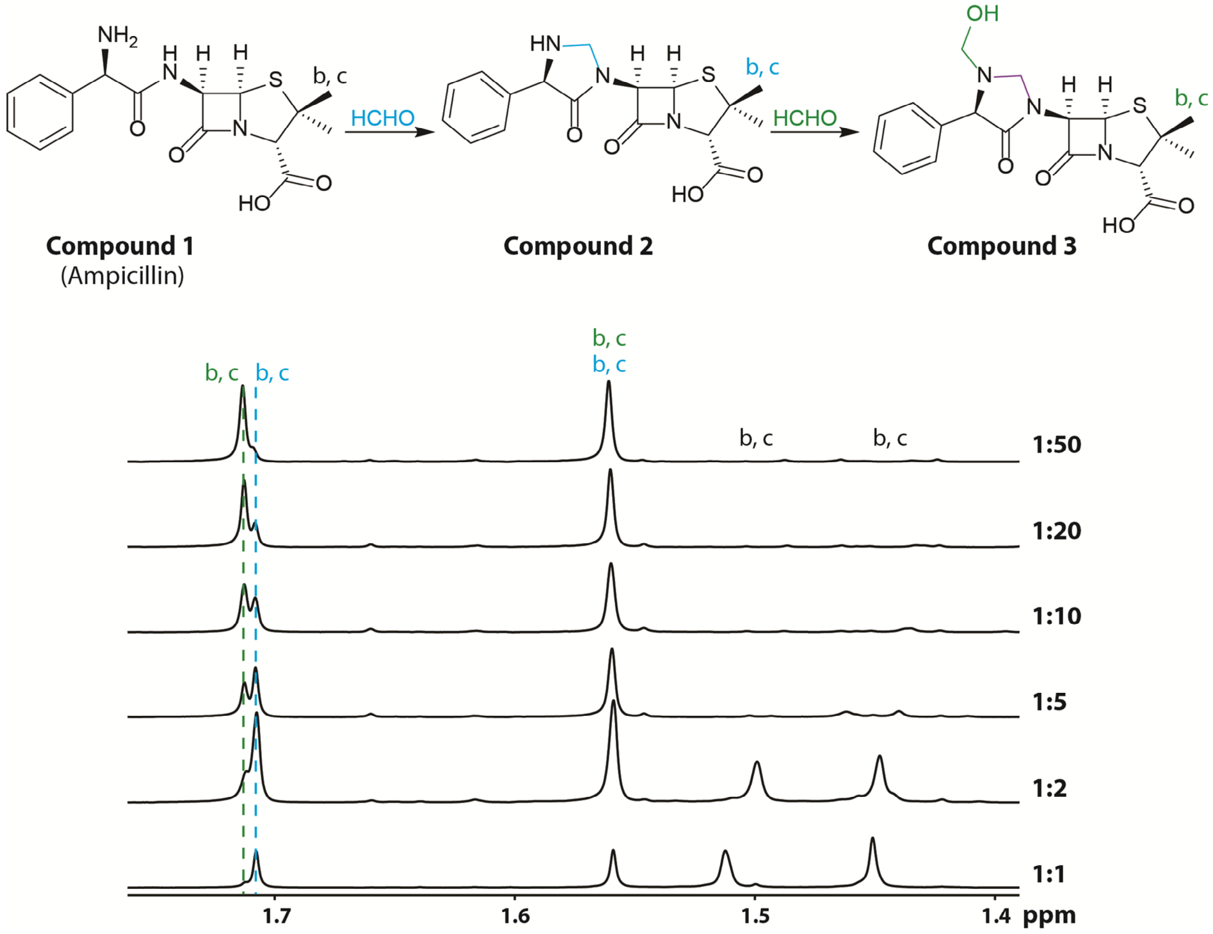

Figure S9 | ^{1}H NMR (700 MHz, D_2O) spectra of the methyl group region after mixing of **1** with different amounts of HCHO (4 h). The two singlet resonances at δ_H ~1.45 and ~1.51 correspond to ampicillin (**1**). The two overlapping singlets at δ_H ~1.708 and 1.713 ppm are assigned to the cyclic aminal adduct **2** (higher field singlet) and product **3** (lower field singlet), respectively. An increased amount of HCHO results in the equilibrium favouring **3**. The singlet at δ_H 1.56 ppm corresponds to coincident resonances, corresponding to one methyl group from **2** and one methyl group from **3**.

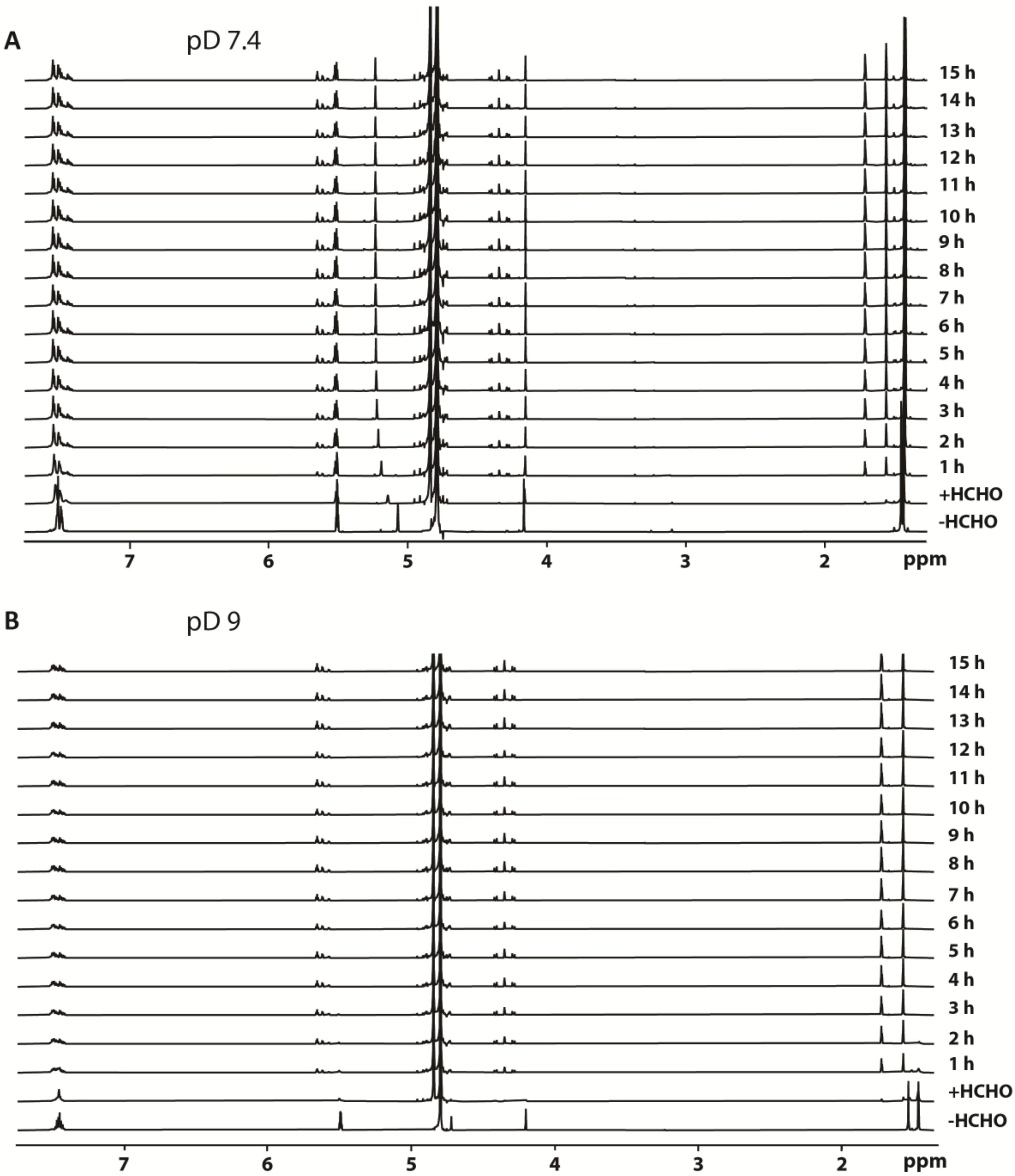

Figure S10 | The reaction of **1** with HCHO to give **2** and **3** as monitored by ^{1}H NMR (700 MHz, D_2O). Note that the reaction is more efficient in basic conditions. The spectra correspond to reactions with a 10-fold excess of HCHO over 15 h at room temperature at pD 7.4 (A) and pD 9 (B).

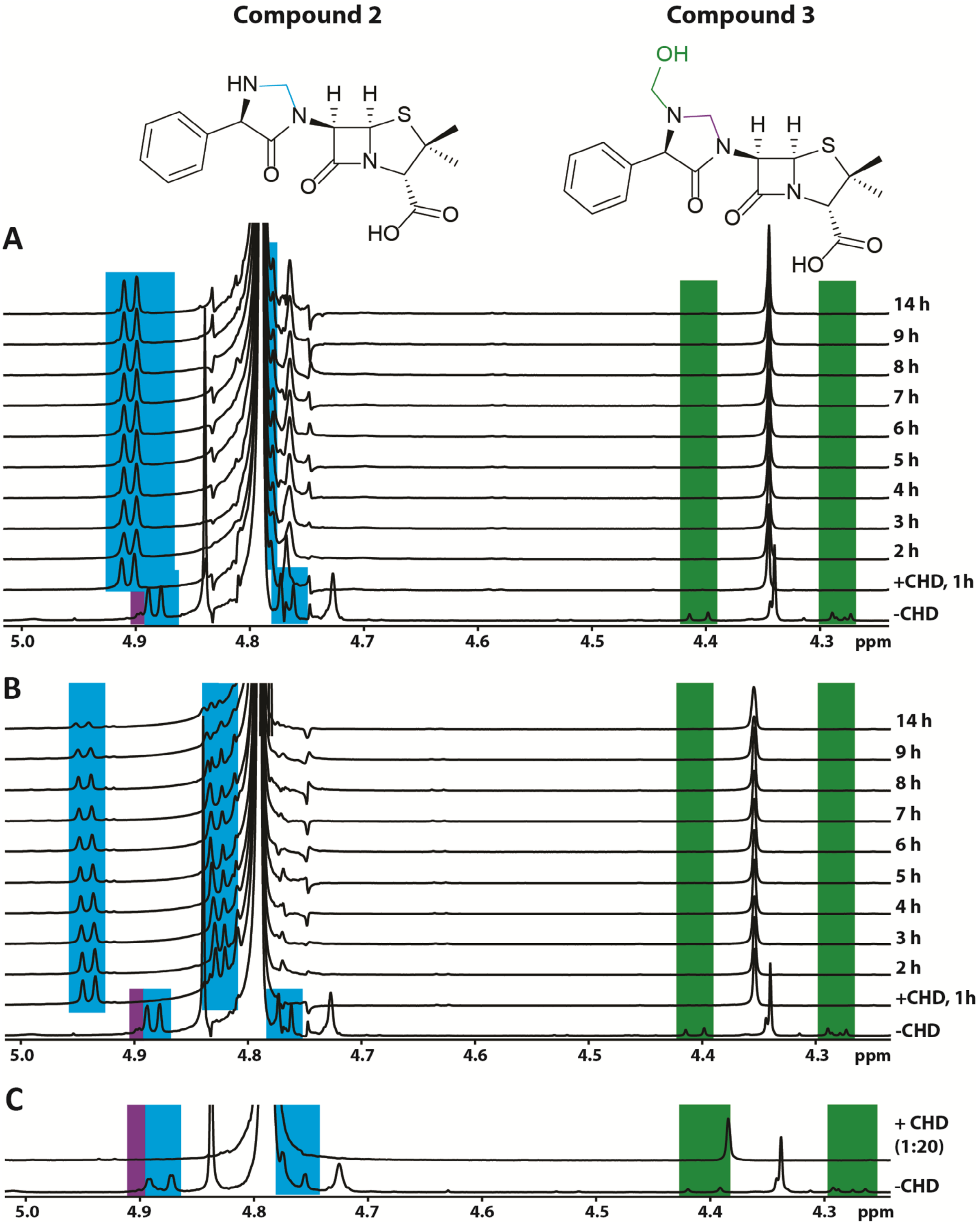

Figure S11 | ^{1}H NMR (700 MHz, D_2O) time-courses showing the degradation of **2** and **3** after addition of 1,3-cyclohexanedione (CHD). **2** and **3** were produced *in situ* by treatment of **1** with a 2-fold excess of HCHO overnight. **A.** 1,3-cyclohexanedione (2:1 relative to HCHO) was added and reaction monitored over 14 h. The signals corresponding to **3** (green, purple) were rapidly lost, whereas those corresponding to the **2** (blue) were more stable. **B.** Increasing the amount of 1,3-cyclohexanedione to 4-fold with respect to HCHO results in slow degradation of the cyclic aminal of **2**. After 14h, ~40% of **2** is still present. **C.** Overlay of ^{1}H spectra (400 MHz, D_2O) before and after 20-fold 1,3-cyclohexanedione treatment shows rapid loss of **2** and **3** and reformation of **1** (Figure S12).

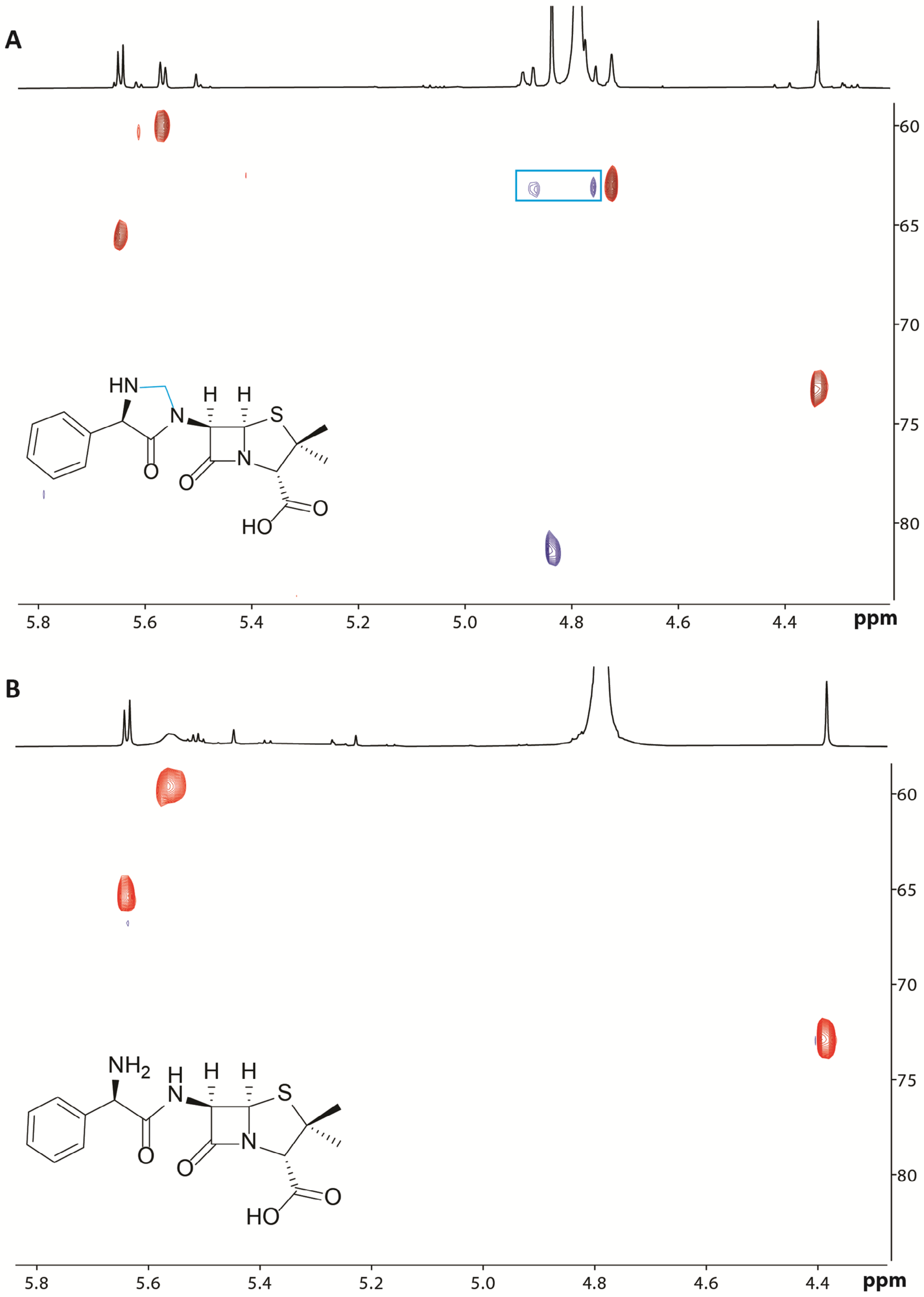

Figure S12 | Stability of the cyclic aminal (**2**) after reaction with a 20-fold excess of 1,3-cyclohexanedione was monitored by ^{1}H-^{13}C-HSQC (400 MHz, D_2O) . **A. 1** was reacted with a 2-fold excess HCHO overnight at room temperature. The presence of a methylene bridge (highlighted in a rectangle) in **2** was confirmed by ^{1}H-^{13}C-HSQC. **B.** The methylene bridge in **2** is removed upon addition of 20-fold excess of 1,3-cyclohexanedione as observed by ^{1}H-^{13}C-HSQC.

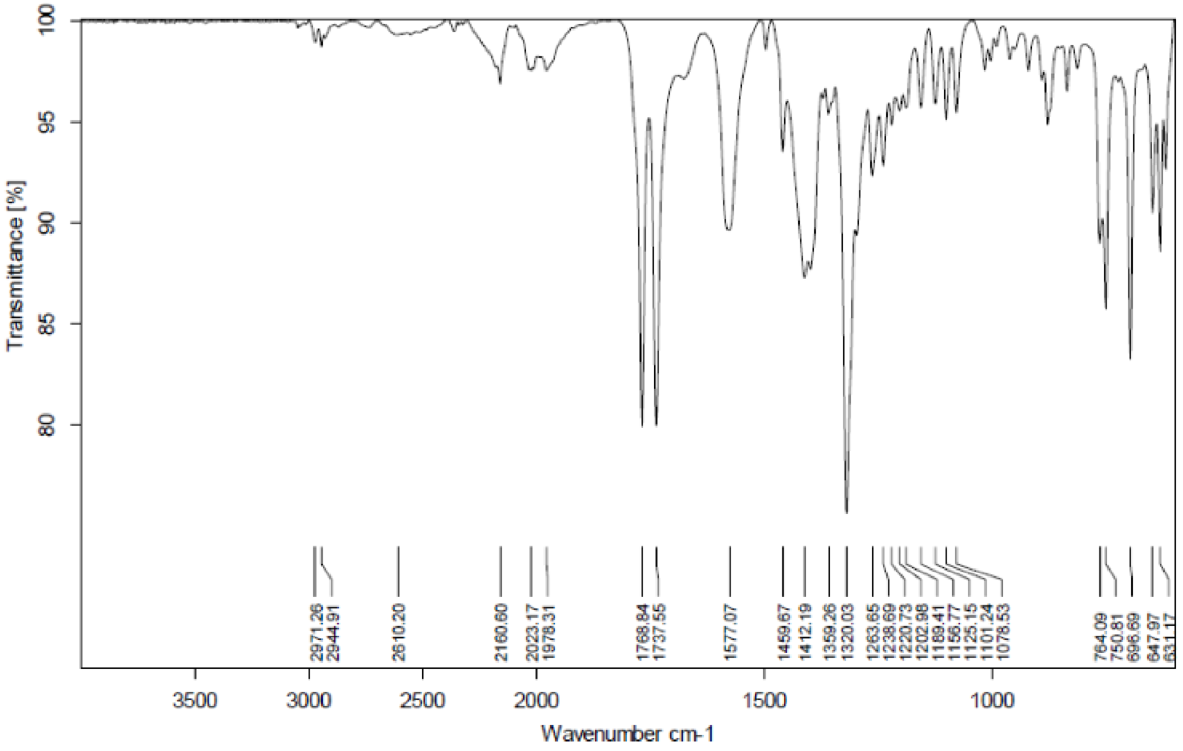

Figure S13 | Infrared analysis of HPLC purified metampicillin ($\tilde{\nu}$ 1769 cm^{-1}, C=O lactam; 1738 cm^{-1}, C=O amide).

References

1. https://www.bruker.com/fileadmin/user_upload/8-PDF-Docs/MagneticResonance/Service_NMR/Acquisition-Processing/acquisition-reference.pdf
